# Systematic Pan-Cancer Analysis of KLRB1 with Prognostic Value and Immunological Activity across Human Tumors

**DOI:** 10.1155/2022/5254911

**Published:** 2022-01-03

**Authors:** Xin Cheng, Yucheng Cao, Xiaowei Wang, Lin Cheng, Yaqiong Liu, Jun Lei, Weijun Peng, Dazun Shi

**Affiliations:** ^1^Department of Integrated Traditional Chinese & Western Medicine, The Second Xiangya Hospital, Central South University, Changsha, China; ^2^The Second Clinical College of Guangzhou University of Chinese Medicine, Guangzhou, Guangdong 510006, China; ^3^Department of Pathology, The Second Xiangya Hospital, Central South University, Changsha, China; ^4^Regenerative Medicine Institute (REMEDI), National University of Ireland Galway, University Road, Galway, Ireland H91 TK33; ^5^Department of Gynecology and Obstetrics, Xiangya Hospital, Central South University, Changsha, Hunan 410008, China

## Abstract

**Introduction:**

KLRB1 is a gene encoding CD161 expressed in NK cells and some T cell subsets. At present, KLRB1 is believed to affect tumorigenesis and development by regulating the cytotoxicity of NK cells in several cancers. However, there is a lack of systematic reviews of KLRB1 in a variety of malignancies.

**Objectives:**

Hence, our research is aimed at providing a relatively comprehensive understanding of the role of KLRB1 in different types of cancer, paving the way for further research on the molecular mechanism and immunotherapy potential of KLRB1.

**Methods:**

In this study, we used relevant public databases, including TCGA (The Cancer Genome Atlas), GEO (Gene Expression Omnibus), CCLE (Cancer Cell Line Encyclopedia), GTEx (Genotype Tissue-Expression), and HPA (Human Protein Atlas), to perform a pan-cancer analysis of KLRB1 across 33 types of cancer. We explored the potential molecular mechanism of KLRB1 in clinical prognosis and tumor immunity from the aspects of gene expression, survival status, clinical phenotype, immune infiltration, immunotherapy response, and chemotherapeutic drug sensitivity.

**Results:**

KLRB1 was downregulated in 13 cancers while upregulated in kidney cancer. Patients with high expression of KLRB1 have a better prognosis in most types of cancer. Moreover, the KLRB1 expression level is related to TMB and MSI and related to various immune signatures of tumor. The expression of KLRB1 can affect tumor immune cell infiltration. KLRB1 expression level can also affect the sensitivity of chemotherapy drugs.

**Conclusions:**

KLRB1 may be a prognostic and immunological biomarker across tumors. At the same time, KLRB1 expression can reflect the sensitivity of cancer patients to chemotherapy drugs. KLRB1 may become a new target for immunotherapy.

## 1. Introduction

Cancer is a significant burden on human lifespan, and its incidence and mortality are increasing rapidly in every country of the world [1]. Therefore, it is essential to identify the molecular mechanisms and the role of specific cancer genes to discover novel therapeutic targets. A molecular pan-cancer analysis can reveal common characteristics and heterogeneity among malignant tumors [2] and will provide a more comprehensive understanding of the molecular pathobiology underlying cancer.

KLRB1 or killer cell lectin-like receptor B1 is a gene encoding CD161, a lectin transmembrane type II receptor expressed on natural killer (NK) cells, CD8+, CD4+, and other T cell subgroups [3–5]. Studies have found that KLRB1 plays a vital role in the differentiation of lymphocytes, especially dendritic cells and monocytes [6]. In addition, CD161 expression can identify NK cells that may contribute to the pathogenesis of inflammatory diseases [7]. In the periphery, the cross-linking of CD161 can increase interferon- (INF-) gamma expression and may inhibit the cytotoxicity of NK cells [8, 9]. Increasing evidence has revealed that the immune functions of KLRB1 have a major impact on the occurrence and development of tumors. A study of the characteristics of tumor immune cells indicated that T/NK cell gene expression is closely related to patient survival in different cancer types [10]. A series of studies on prognostic signatures related to tumor immunity have pointed out that KLRB1 is a crucial gene affecting prognoses in lung adenocarcinoma [11], liver cancer [12], and breast cancer [13]. Other reports have shown that KLRB1 can be applied as a prognostic factor in esophageal squamous cell carcinoma [14] and non-small-cell lung cancer [15]. At present, cancer immunotherapy has received widespread attention and has been successfully applied clinically [16], but there are still many patients who achieve limited or no response to immunotherapy [17]. Therefore, it is very urgent to explore tumor immunity and identify new immunotherapy targets. Blocking the LLT1-CD161 signaling pathway in lung cancer [15], prostate cancer [18], and diffuse glioma [19] can inhibit the activity of NK cells and may become a breakthrough agent in immunotherapy.

However, the current research on the value of KLRB1 in tumor immunity and prognosis has concerned only a small number of cancers. There is no understanding of the shared or differing roles of KLRB1 in different types of cancer from the perspective of pan-cancer. Therefore, our study conducted a pan-cancer analysis of KLRB1 in 33 cancers extracting data from multiple databases, including datasets from The Cancer Genome Atlas (TCGA) and the Gene Expression Omnibus (GEO). We identified significant differences in the expression of the KLRB1 gene between normal and tumor specimens in 15 cancers. Our study also clarified that the high expression of KLRB1 in most tumors is associated with favorable outcomes. Moreover, we studied the influence of KLRB1 expression on the tumor microenvironment (TME) and explored the relationship between KLRB1 and various immune biomarkers. Our results indicated that KLRB1 can be used as a prognostic biomarker and can predict immune status. We also discovered the potential value of KLRB1 in predicting chemotherapeutic drug sensitivity. Our research initially revealed the critical role of KLRB1 in predicting the prognosis of patients and tumor immunity, data which can be used as a reference for immunotherapy new targets and as a potential choice for clinical treatment options.

## 2. Methods

### 2.1. Data Collection

RNA sequence data, clinically relevant data, and somatic mutation data of 33 tumors deriving from TCGA were downloaded from the UCSC Xena (https://xena.ucsc.edu/). Gene expression data of normal tissues using a dataset from the GTEx database (https://commonfund.nih.gov/GTEx) and the RNA sequencing data of different cancer cell lines were obtained from the CCLE database (https://portals.broadinstitute.org/ccle/). Moreover, we also used gene expression profiles from the GEO to compare expression between normal tissues and tumor tissues (https://www.ncbi.nlm.nih.gov/geo/). The selected datasets included GSE13507, GSE139038, GSE37128, GSE25093, GSE40435, GSE121248, GSE10072, GSE33532, GSE54129, GSE87211, and GSE57545. The list of the tumor datasets used in this study is shown in Supplementary Table S1.

The Human Protein Atlas (HPA) is a database containing maps of all human proteins in cells, tissues, and organs. We downloaded immunohistochemistry (IHC) images of normal tissues and cancer tissues from The Tissue Atlas and The Pathology Atlas (http://www.proteinatlas.org/).

### 2.2. Survival Analysis

To assess the role of KLRB1 in prognosis, we analyzed the relationship between KLRB1 expression and prognosis indicators in each tumor type, including overall survival (OS), disease-specific survival (DSS), disease-free interval (DFI), and progression-free interval (PFI). This study performed a univariate Cox regression analysis, and a *p* value less than 0.05 was considered statistically significant. Kaplan–Meier (K-M) curves were also used to explore the impact of KLRB1 expression on survival time, and a log-rank test was performed. A *p* value < 0.05 was statistically significant.

### 2.3. Evaluation of Tumor Mutation Burden and Tumor Microsatellite Instability in Tumors

Based on the somatic mutation data obtained from TCGA, we calculated the tumor mutation burden (TMB) score (the total number of all mutations except silent mutations) for each patient and determined the tumor microsatellite instability (MSI) score of TCGA. We also explored the correlation between KLRB1 and mismatch repair (MMR) genes, which can correct DNA replication errors, and how their expression affects the frequency of gene mutations [20]. The MMR genes included in this study were MLH1, MSH2, MSH6, PMS2, and EpCAM.

### 2.4. Evaluation of the Tumor Microenvironment and Immune Cell Infiltration

We used the ESTIMATE approach [21] to determine the degree of penetration of immune cells and stromal cells in each tumor sample and obtained an immune score and stromal score to represent each tumor's immune status. Moreover, we used two methods to evaluate the degree of immune cell infiltration in tumor samples. The first was CIBESORT [22], which allowed to calculate the abundance scores of 22 immune cells, and the other involved using single-sample gene set enrichment analysis (ssGSEA) and marker genes to identify T cells, macrophages, and B cells. The marker genes of the three cell types are listed in Supplementary Table S2.

### 2.5. Evaluation of Immune Signatures in Each Tumor Sample

We selected four immune-related indicators to evaluate the immunological characteristics of each sample, including HLA, TILs, immune cytolytic activity (CYT), and IFN response. We analyzed relevant gene sets (Table S2) from previous studies [23–25] and then performed ssGSEA to quantify the immune status of each sample.

To determine the ability of KLRB1 to predict immunity, we calculated the glycolysis score [26] of each tumor sample. The score was obtained by ssGSEA using related gene sets based on a previous study [26] (Table S2). We compared the correlation between three indicators KLRB1, TMB, glycolysis score, and tumor immune activity assessed by the immune score and CYT.

To evaluate the role of KLRB1 in immunotherapy, we performed ssGSEA using the immune checkpoint-related gene set for each tumor sample. We then explored the correlation between KLRB1 expression and the scores of immune checkpoint genes and determined whether there were differences in the scores of immune checkpoints between patients with high and low expression of KLRB1.

### 2.6. Enrichment Analysis

To investigate the biological significance of KLRB1, we performed GSEA. The Gene Ontology (GO) and Kyoto Encyclopedia of Genes and Genomes (KEGG) gene sets used were c5.go.v7.2.symbols and c2.cp.kegg.v7.2.symbols. A normalized *p* value of <0.05 was considered statistically significant.

### 2.7. Chemotherapy Drug Sensitivity

To explore the role of KLRB1 in the chemotherapy of cancer patients, we calculated the drug sensitivity of each cancer patient using the R software package “pRRophetic” and performed a difference analysis comparing high and low KLRB1 expression groups.

### 2.8. Statistical Analysis

Log_2_ transformation was performed to normalize the downloaded TCGA and GEO raw data. The Wilcoxon rank-sum test was used to analyze differences between the two samples, and *p* < 0.05 was considered statistically significant. Spearman's method was used to analyze two-variable correlations, and a *p* value < 0.05 indicated the two variables are significantly correlated. All statistical analyses in this study were performed using R software (version 4.0.2).

## 3. Results

### 3.1. KLRB1 Was Differentially Expressed in Normal and Tumor Samples

**Figure 1:**
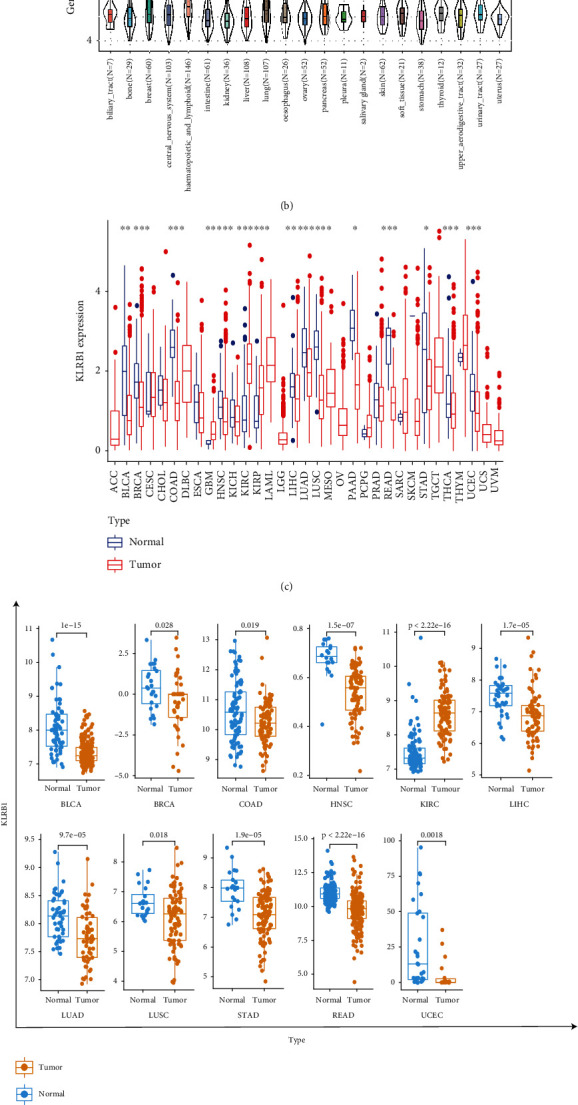
Expression of KLRB1 in normal and tumor tissues. (a) KLRB1 expression across 31 regular tissues and (b) 21 tumor cell lines. (c) Differences in KLRB1 expression between normal and tumor tissues in 33 tumor types from TCGA. *p* values are based on the Wilcoxon rank-sum test. (d) Significant difference of KLRB1 expression between normal and tumor tissues in 11 tumors based on GEO datasets. *p* values are based on the Wilcoxon rank-sum test. ^∗^*p* < 0.05, ^∗∗^*p* < 0.01, and ^∗∗∗^*p* < 0.001. (e) Immunohistochemistry images. The expression of KLRB1 protein in tumor tissues is significantly lower than that in normal tissues across 6 types of cancer.

We used the GTEx dataset to analyze the normal expression of the KLRB1 gene in tissues (Figure 1(a)). KLRB1 was expressed at higher levels in the spleen, small intestine, and lung tissues, but at a lower level in other normal tissues. Based on the CCLE dataset, expression levels of KLRB1 in different cancer cell lines are shown in Figure 1(b). KLRB1 was more evenly expressed in different tumors.

We compared KLRB1 expression levels between tumor and normal tissues, according to TCGA database. Among the 33 cancers, 15 cancers were found to have a statistically significant differential expression of KLRB1 compared with normal samples (Figure 1(c)). Moreover, compared with normal tissues, KLRB1 expression was lower in bladder urothelial carcinoma (BLCA), breast invasive carcinoma (BRCA), colon adenocarcinoma (COAD), head and neck squamous cell carcinoma (HNSC), liver hepatocellular carcinoma (LIHC), lung adenocarcinoma (LUAD), lung squamous cell carcinoma (LUSC), pancreatic adenocarcinoma (PAAD), rectum adenocarcinoma (READ), stomach adenocarcinoma (STAD), thyroid carcinoma (THCA), and uterine corpus endometrial carcinoma (UCEC). In contrast, KLRB1 was highly expressed in glioblastoma multiforme (GBM), kidney renal clear cell carcinoma (KIRC), and kidney renal papillary cell carcinoma (KIRP). We verified the differences in KLRB1 expression between normal and tumor samples in the GEO database. Eleven cancers showed significant differences in KLRB1 expression between normal and tumor samples (Figure 1(d)). Consistent with TCGA results, except for KIRP, KLRB1 showed low expression in the other ten cancers.

Furthermore, to verify the protein expression of KLRB1, we obtained the IHC results from the HPA database. As shown in Figure 1(e), normal breast, liver, lung, pancreas, and stomach tissues showed moderate KLRB1 IHC staining, while in tumor tissues staining was even less marked. These results are consistent with their gene expression levels.

### 3.2. KLRB1 Expression Was Significantly Related to Prognosis in Various Cancers

**Figure 2:**
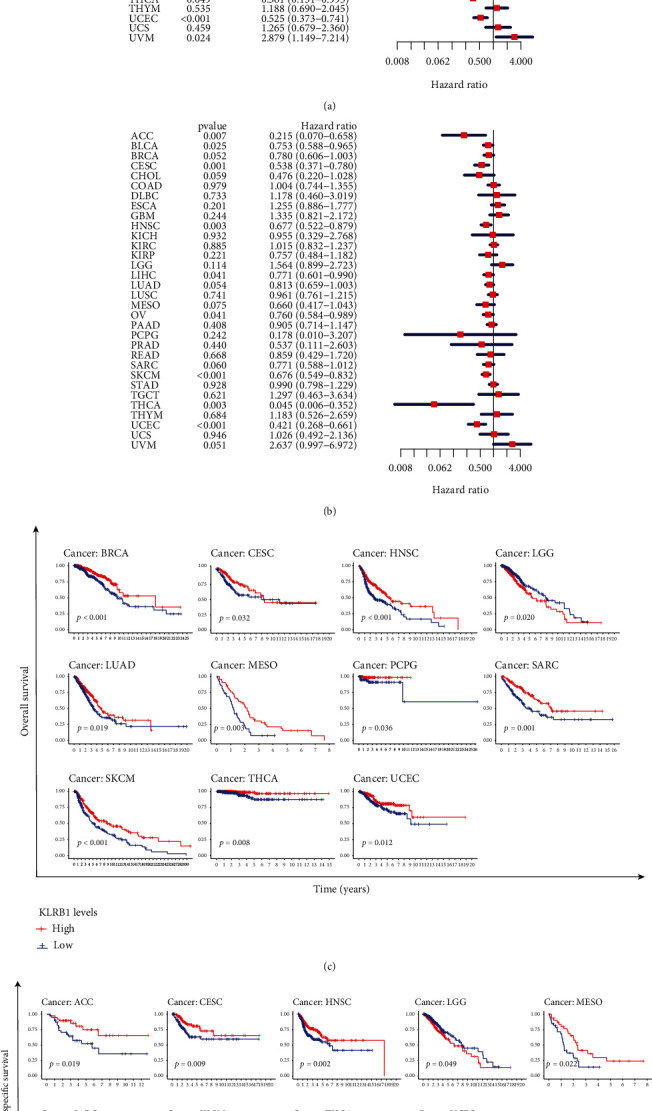
The relationship between KLRB1 expression and patient prognosis. (a) Univariate Cox regression of KLRB1 expression for overall survival (OS) in 33 cancers. (b) Univariate Cox regression of KLRB1 expression for disease-specific survival (DSS) in 33 cancers. (c) The Kaplan–Meier curves of OS in the low- and high-expression KLRB1 groups. (d) The Kaplan–Meier curves of DSS in the low- and high-expression KLRB1 groups.

To evaluate the prognostic value of KLRB1 in different tumors, we selected four prognostic indicators, OS, DSS, DFI, and PFI, and performed a Cox regression analysis drawing Kaplan–Meier curves. As shown in Figure 2(a), high expression of KLRB1 was associated with a better prognosis in most cancers, including adrenocortical carcinoma (ACC) (*p* = 0.009), BRCA (*p* < 0.001), cervical squamous cell carcinoma and endocervical adenocarcinoma (CESC, *p* < 0.001), HNSC (*p* < 0.001), LIHC (*p* = 0.004), LUAD (*p* = 0.002), mesothelioma (MESO, *p* = 0.015), ovarian serous cystadenocarcinoma (OV, *p* = 0.024), sarcoma (SARC, *p* = 0.010), skin cutaneous melanoma (SKCM, *p* < 0.001), THCA (*p* = 0.049), and UCEC (*p* < 0.001), while KLRB1 was a high-risk gene in uveal melanoma (UVM, *p* = 0.009).

The Kaplan–Meier curves for OS also showed similar results that patients with low levels of KLRB1 had longer survival times in BRCA (*p* < 0.001), CESC (*p* = 0.032), HNSC (*p* < 0.001), LUAD (*p* = 0.019), MESO (*p* = 0.003), pheochromocytoma and paraganglioma (PCPG) (*p* = 0.036), SARC (*p* = 0.001), SKCM (*p* < 0.001), THCA (*p* = 0.008), and UCEC (*p* = 0.012), while patients with low levels of KLRB1 experienced more favorable outcomes in LGG (*p* = 0.020) (Figure 2(c)).

In terms of DSS, KLRB1 was a protective gene in ACC (*p* = 0.007), BLCA (*p* = 0.025), CESC (*p* = 0.001), HNSC (*p* = 0.003), LIHC (*p* = 0.041), OV (*p* = 0.041), SKCM (*p* < 0.001), THCA (*p* = 0.003), and UCEC (*p* < 0.001) (Figure 2(b)). Kaplan–Meier curves also revealed that except for LGG (*p* = 0.049), higher levels of KLRB1 expression were associated with better outcomes in ACC (*p* = 0.019), CESC (*p* = 0.009), HNSC (*p* = 0.002), MESO (*p* = 0.022), SARC (*p* = 0.029), SKCM (*p* < 0.001), THCA (*p* = 0.001), and UCEC (*p* = 0.001) (Figure 2(d)).

Cox regression analysis (Figure S1A) showed that KLRB1 was a low-risk factor associated with PFI in BLCA (*p* = 0.021), BRCA (*p* = 0.031), CESC (*p* = 0.025), cholangiocarcinoma (CHOL, *p* = 0.047), LIHC (*p* = 0.006), and UCEC (*p* = 0.033). Kaplan–Meier analysis (Figure S1C) showed that patients with high levels of KLRB1 expression had better outcomes in COAD (*p* = 0.016), CHOL (*p* = 0.008), and LICH (*p* = 0.014).

Moreover, KLRB1 was also significantly associated with PFI in ACC (*p* = 0.001), BLCA (*p* = 0.023), BRCA (*p* = 0.008), CESC (*p* = 0.001), CHOL (*p* = 0.017), HNSC (*p* = 0.018), LIHC (*p* = 0.016), MESO (*p* = 0.041), SKCM (*p* = 0.027), and UCEC (*p* < 0.001) (Figure S1B). Kaplan–Meier curves also illustrated that KLRB1 was a low-risk factor associated with PFI in ACC (*p* < 0.001), BRCA (*p* = 0.036), CESC (*p* = 0.017), CHOL (*p* = 0.022), HNSC (*p* = 0.008), LIHC (*p* = 0.029), MESO (*p* = 0.009), SKCM (*p* = 0.037), and UCEC (*p* = 0.011) (Figure S1D).

### 3.3. KLRB1 Expression Affected the Clinical Phenotype of Patients with Various Cancers

**Figure 3:**
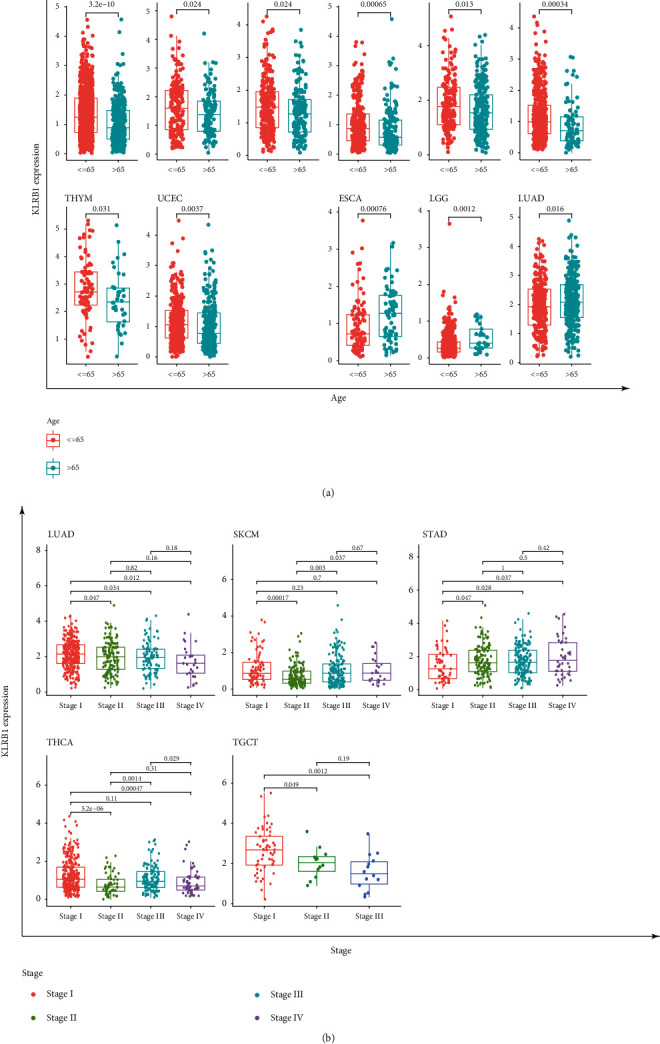
Relationship between KLRB1 expression and clinical phenotypes in different cancers. Association between KLRB1 expression and (a) age and (b) stage.

First, we explored the relationship between KLRB1 expression and age. The patients were divided into the low-age group and the high-age group with age 65 years as the cutoff value. As shown in Figure 3(a), KLRB1 was expressed at a higher level in elderly patients in BRCA, KIRP, LIHC, SKCM, STAD, THCA, thymoma (THYM), and UCEC, while the expression of KLRB1 in elderly patients was significantly lower than that in the younger age group in esophageal carcinoma (ESCA), LGG, and LUAD.

Moreover, in LUAD, SKCM, STAD, THCA, and testicular germ cell tumors (TGCT), KLRB1 expression was also significantly correlated with tumor stage. It is noteworthy that the most significant differences occurred between the first and second stages (Figure 3(b)), and except for the positive correlation in STAD, the expression of KLRB1 decreased with increasing tumor stages. Interestingly, differences in KLRB1 expression levels between high-stage tumors were minimal and were not statistically significant in most cases.

### 3.4. KLRB1 Expression Levels Were Associated with TMB, MSI, and MMR Genes

**Figure 4:**
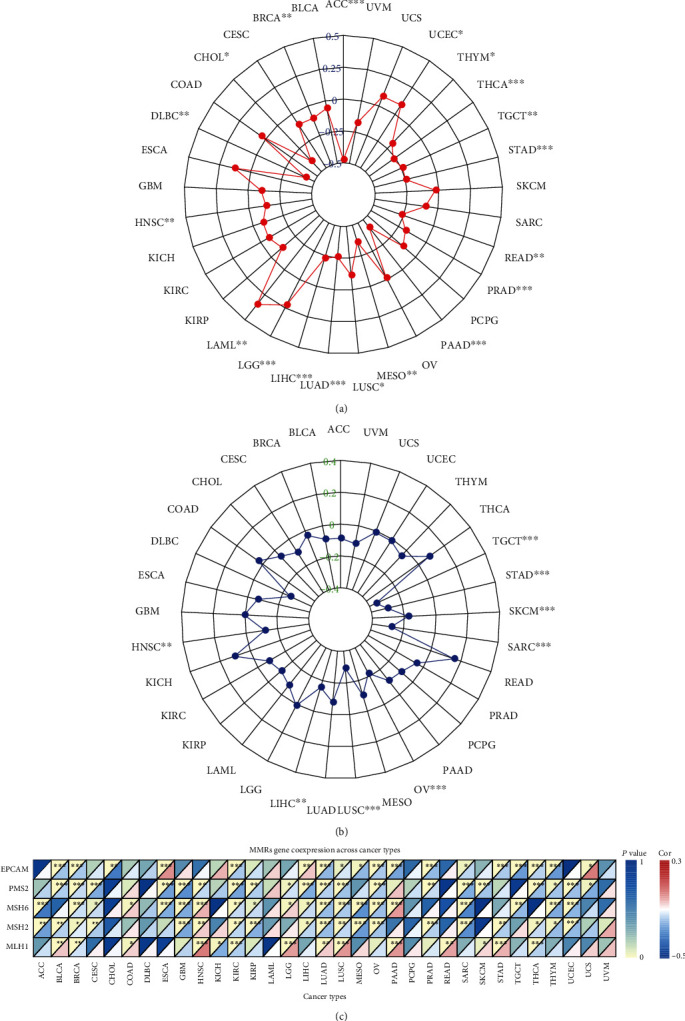
The relationship between KLRB1 expression and the TMB and MSI and MMR gene expression. (a) Relationship between KLRB1 and TMB (red curve of the radar chart indicates the correlation coefficient, and blue numbers indicate the range). (b) Relationship between KLRB1 and MSI (blue curve of the radar chart indicates the correlation coefficient, and green numbers indicate the range). (c) Relationship between KLRB1 expression and MMR genes (for each pair, top left triangle represents the *p* value, and bottom right triangle indicates the correlation coefficient). ^∗^*p* < 0.05, ^∗∗^*p* < 0.01, and ^∗∗∗^*p* < 0.001.

Next, we explored the correlation between KLRB1 with TMB and MSI, two indicators that can affect the sensitivity to immune checkpoint inhibitor therapy. We performed coexpression analysis between MMR genes (MLH1, MSH2, MSH6, PMS2, and EpCAM) and KLRB1 genes. The results showed that in 17 tumors such as BRCA, LIHC, LUAD, and STAD, the expression of KLRB1 was positively correlated with TMB, but the opposite was true in acute myeloid leukemia (LAML) and brain lower-grade glioma (LGG) (Figure 4(a)). There was a negative correlation between the expression of KLRB1 and MSI in 8 tumors, including LIHC, LUSC, and other cancers (Figure 4(b)). Detailed results were shown in Supplementary Table S3.  Figure 4(c) shows the coexpression relationship between KLRB1 and different MMR genes. MLH1, MSH2, MSH6, PMS2, and KLRB1 expression levels were significantly negatively correlated in most cancers, while EpCAM and KLRB1 expression levels were positively correlated.

### 3.5. KLRB1 Impacted on the Tumor Microenvironment

To evaluate the tumor microenvironment (TME) of the samples, we used the ESTIMATE algorithm to score the immune and stromal cells of each sample. Moreover, we analyzed the relationship between the levels of expression of KLRB1 and both scores. Except for LAML, in the remaining 32 cancers, the expression level of KLRB1 was positively correlated with the immune score. In 25 cancers, including breast, bladder, liver, and lung cancer, the expression of KLRB1 was also positively correlated with the stromal score. Figure S2 shows the top 6 tumors with the strongest correlation. The detailed results are reported in Supplementary Table S4.

**Figure 5:**
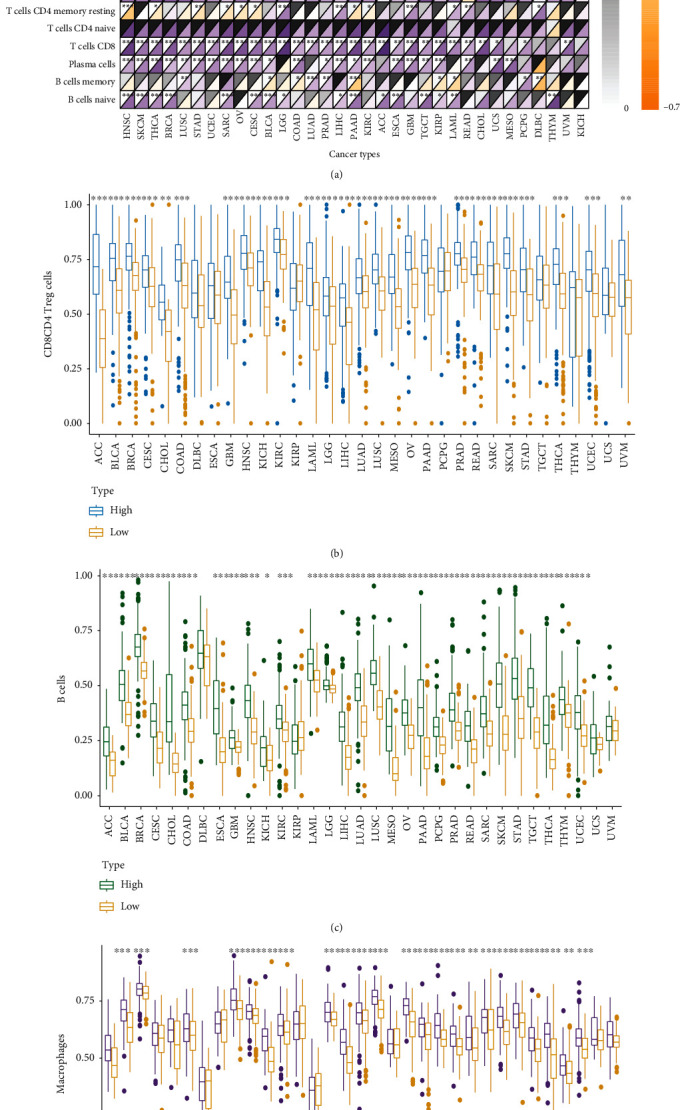
Differences in KLRB1 expression are associated with differences in distribution of immune cell infiltration. (a) The relationship between KLRB1 expression and the degree of immune cell infiltration in different cancers (evaluated using the CIBERSORT method). For each small grid, the upper left triangle represents the *p* value, and the lower right triangle represents the correlation coefficient. Distribution infiltration of (b) CD8+ T and CD4+ T cells, (c) B cells, and (d) macrophages (using marker genes' expression analysis) stratified by KLRB1 high- and low-expression groups. ^∗^*p* < 0.05, ^∗∗^*p* < 0.01, and ^∗∗∗^*p* < 0.001.

Subsequently, we explored the expression of immune cells in the TME using two methods, CIBERSORT and ssGSEA, to evaluate the degree of infiltration of various cells. The results showed that in most tumors, the expression of KLRB1 was associated with the degree of immune cell infiltration (Figure 5(a)). With increasing KLRB1 expression, the infiltration of M0 macrophages and M2 macrophages decreased, and the infiltration of lymphoid cells such as T cells and B increased. Cancer patients with higher expression of KLRB1 had higher levels of T cells (Figure 5(b)) and B cells (Figure 5(c)) infiltrated in the tumor microenvironment. Patients with high expression of KLRB1 appeared to have higher levels of macrophages in the tumor (Figure 5(d)).

### 3.6. The Potential Value of KLRB1 in Predicting Tumor Immunity

**Figure 6:**
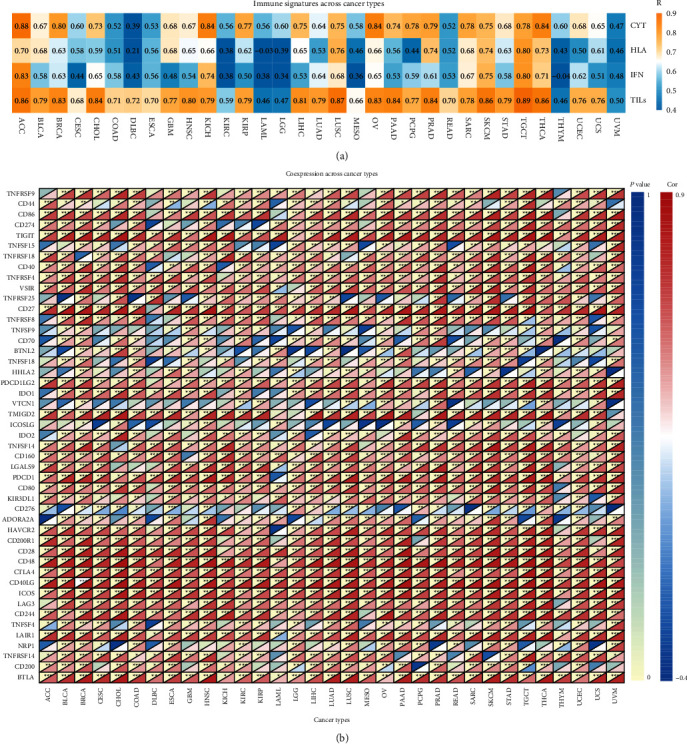
The relationship between KLRB1 and immune-related genes. (a) KLRB1 expression is associated with various immune signatures, among the 33 cancer types in TCGA, including immunocytolytic activity (CYT), HLA, interferon (IFN), and TILs. (b) The heat map of coexpression between KLRB1 and immune-related genes. For each small grid, the upper left triangle represents the *p* value, and the lower right triangle represents the correlation coefficient. ^∗^*p* < 0.05, ^∗∗^*p* < 0.01, and ^∗∗∗^*p* < 0.001.

To further study the effects of KLRB1 on tumor immunity, we analyzed the correlation between KLRB1 expression and four immune indicators (cytolytic activity, HLA expression, IFN response, and TILs). In 33 cancers, the expression of KLRB1 was significantly correlated with these immune signatures, and most were positively correlated (Supplementary Table S5). It is worth noting that among the four indicators, the TILs showed the most significant correlation with KLRB1 (Figure 6(a)).

We also analyzed the relationship between KLRB1 and other immune-related genes, having immune activation or suppression effects. As shown in Figure 6(b), KLRB1 is significantly associated with almost all immune-related genes among 33 cancers; KLRB1 and most of these genes were positively correlated.

**Figure 7:**
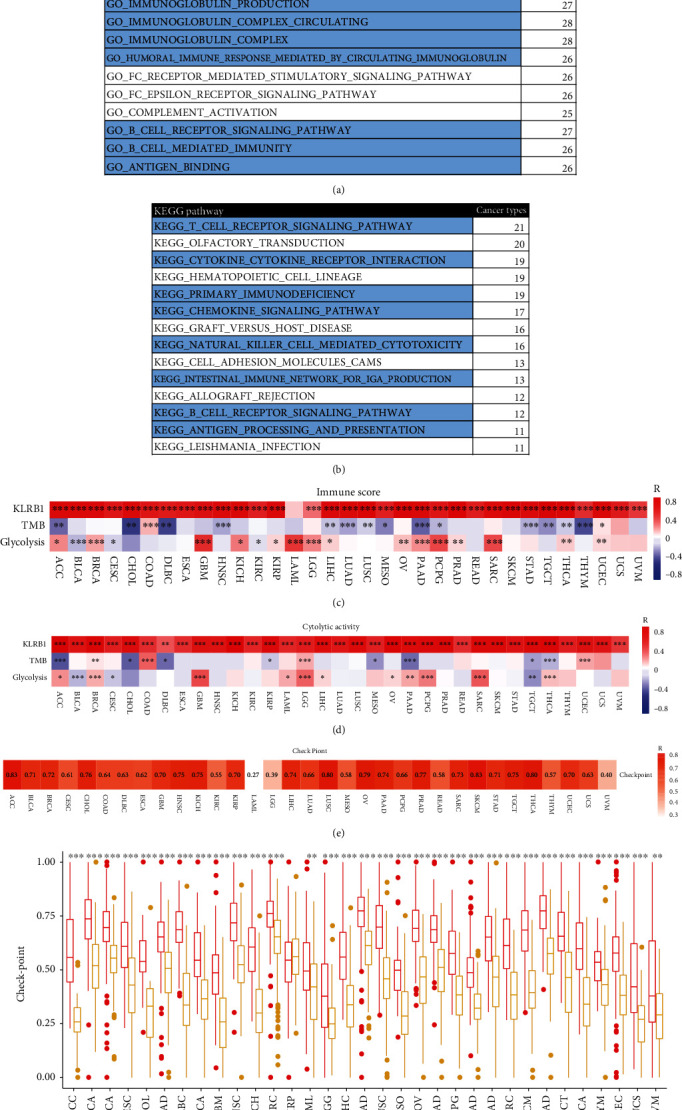
KLRB1 can be used as a potential indicator of response to immunotherapy. (a) Enriched Gene Ontology (GO) biological function of KLRB1 across various cancers. (b) Enriched Kyoto Encyclopedia of Genes and Genomes (KEGG) pathways of KLRB1 across various cancers. Comparison of the correlation between KLRB1 expression, TMB, and glycolytic activity and (c) the immune score and (d) cytolytic activity. (e) Correlation between KLRB1 and the expression of checkpoint genes in 33 cancers. (f) The distribution of the expression level of checkpoint genes stratified by high and low KLRB1 expression for 33 cancers. ^∗^*p* < 0.05, ^∗∗^*p* < 0.01, and ^∗∗∗^*p* < 0.001.

Next, to explore the possible mechanisms relative to the involvement of KLRB1 in tumor immunity, we determined its biological functions using GO and the KEGG pathway enrichment for 33 cancer types through GSEA. KLRB1 played immune-related biological effects in most tumors and was enriched in multiple immune-related pathways, including various immune cell activities, immune responses, antigen processing and presentation, and chemokine signaling pathways (Figures 7(a) and 7(b)). This result suggested that KLRB1 may play a key role in cancer by affecting these signaling pathways and immune cell functions.

### 3.7. KLRB1 Potentially Affects Immunotherapy

Given the role of KLRB1 in immunity, we further studied whether KLRB1 is relevant for immunotherapy. First, we evaluated the ability of KLRB1 to predict tumor immune characteristics, including immune score and cytolytic activity, and compared it with the TMB and glycolytic activity [26]. Except for LAML, KLRB1 showed a high potential for predicting the immune response in all 33 cancers. For both the immune score and CYT, KLRB1 and glycolysis were positively correlated, while the TMB was negatively correlated with the immune score. Of note, KLRB1 showed a stronger correlation (Figures 7(c) and 7(d), Supplementary Table S6).

Next, we obtained the immune checkpoint gene expression score for each sample according to the ssGSEA algorithm and analyzed its correlation with KLRB1 expression. Interestingly, in the 33 cancers evaluated, there was a significant positive correlation between KLRB1 and the checkpoint gene score (Figure 7(e)). For patients with either high or low expression of KLRB1, the expression of immune checkpoint genes was also significantly different, as patients with higher expression of KLRB1 had higher levels of immune checkpoint gene expression across 32 cancers (Figure 7(f)). These results suggested that KLRB1 may serve as a new immunotherapy target and was associated with the response to immunotherapy.

### 3.8. KLRB1 Has a Potential Guiding Role for Chemotherapy Drugs

**Figure 8:**
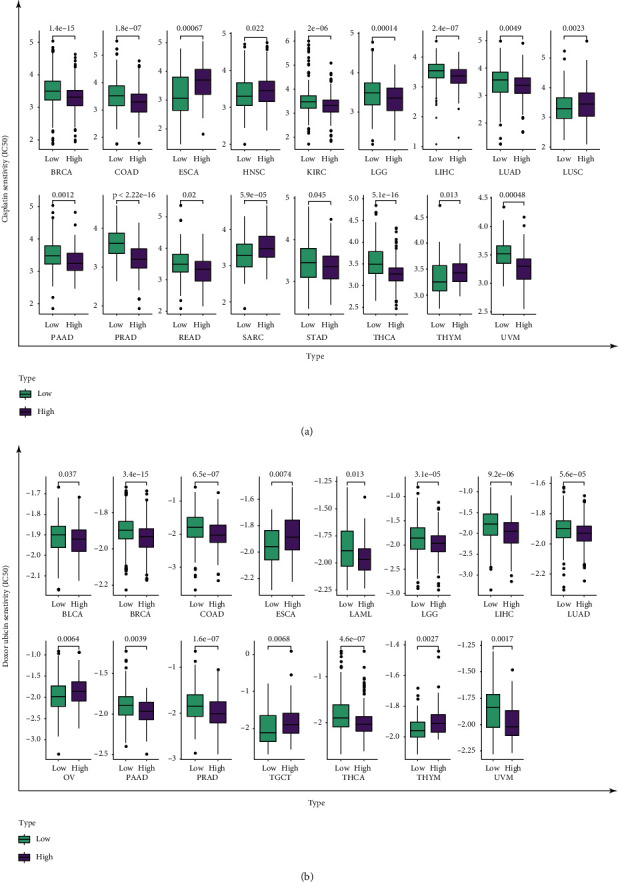
The correlation between KLRB1 expression and chemotherapeutic drug sensitivity. (a) Correlation between KLRB1 expression and cisplatin sensitivity. (b) Correlation between KLRB1 expression and doxorubicin sensitivity.

Chemotherapy is currently the primary treatment method for cancer patients, but the efficacy of chemotherapy varies significantly due to an individual patient's sensitivity to different chemotherapeutic agents [27]. Thus, it is essential to identify a biomarker that can predict drug sensitivity. To this end, we analyzed potential differences in the sensitivity of patients with high and low KLRB1 expression to commonly used chemotherapy drugs (cisplatin and doxorubicin). For cisplatin, in 17 cancers, the expression of KLRB1 variably affected the patients' sensitivity to the drug (Figure 8(a)). For example, ESCA and four tumors with high KLRB1 expression would likely respond better to cisplatin, while 12 tumors, including BRCA with low expression, had higher sensitivity to the drug. For doxorubicin, 15 types of cancer patients with different expression levels of KLRB1 were predicted to exhibit different therapeutic effects (Figure 8(b)). In 4 tumors, including ESCA, the expression of KLRB1 was positively correlated with drug sensitivity, while in 11 tumors, including BLCA, patients having low expression of KLRB1 would achieve a better response to chemotherapy.

## 4. Discussion

Recently, several reports have shown that the KLRB1 gene and its coded protein CD161 play an essential role in tumor immunity [15, 19, 28]. However, the role of KLRB1 in tumorigenesis and tumor development remains elusive from the perspective across multiple cancers. Thus, we conducted a comprehensive analysis of the KLRB1 gene in 33 different tumors based on information extracted from various databases, including data relative to gene expression, prognostic value, immune microenvironment, and response to treatment.

In our study, data from multiple databases demonstrated that KLRB1 was downregulated at both the gene and protein levels in most tumors, which is consistent with the results from a previous study [29]. Interestingly, the expression of KLRB1 in kidney cancer tissues (KIRC and KIRP) showed the opposite results. A previous study has reported that KLRB1 is often associated with a good prognosis [30]. Our results also confirmed that patients with higher expression of KLRB1 achieve longer survival in different cancers including breast cancer, melanoma, and thyroid cancer. Unlike these cancers, patients with low-grade brain gliomas having high KLRB1 expression exhibit a worse prognosis. For patients with hepatocellular carcinoma, the coexpression of CD161 and IL-7R enhances IL-2, TNF-*α*, and perforin expression which improve prognosis [28]. In addition, we also showed that patients with liver cancer expressing KLRB1 had a longer PFI. Recent studies on oropharyngeal squamous cell carcinoma have revealed that the downregulation of CD161 can lead to the immune escape of cancer cells [31], while IL-17 and IFN-*γ* produced by CD161+ T cells can reduce tumor burden and improve OS [32]. Moreover, another study has reported that KLRB1 is also associated with a favorable outcome in non-small-cell lung cancer [15], which is consistent with our findings. We also explored the relationship between the patient's clinical phenotype and KLRB1 expression. It is noteworthy that KLRB1 expression is significantly correlated with age in 11 cancers, which may contribute to guide clinical treatment options and drug selection. Our study also found that the higher the tumor stage, the lower the KLRB1 expression level in LUAD, SKCM, THCA, and TGCT. The above results all indicate that KLRB1 can be used as a prognostic-related predictor in different cancers and may have a functional role in a variety of tumors.

Next, we explored the correlation between KLRB1 and the biomarkers TMB and MSI that are closely related to tumor immunity and immunotherapy response [33, 34]. We showed that the expression of KLRB1 could affect the expression of MMR genes, thereby influencing gene instability, leading to changes in TMB and MSI. Our findings showed that the expression of KLRB1 in 19 cancers was significantly correlated with TMB, and most were negatively correlated. In 8 cancers, correlations between KLRB1 and MSI were also negative. Based on the available evidence, we speculate that patients with low KLRB1 expression and high TMB and MSI may achieve better responses to immune checkpoint blocking therapy.

The role of TME in the process of tumor occurrence and development has attracted increasing attention. The degree of infiltration of tumor immune-related cells can determine the prognosis of tumor patients and may affect a patient's response to immunotherapy [35–37]. In particular, monocytes and M2 macrophages promote cancer development and are related to poor prognosis [38], while M1 macrophages and dendritic cells enhance the antitumor activity and are associated with good prognosis [39]. Our results indicated that the expression of KLRB1 was negatively correlated with the degree of infiltration of cancer-promoting myeloid cells, while it was positively correlated with the degree of infiltration of tumor suppressor myeloid cells. These results indicated that KLRB1 could play a role in inhibiting cancer, further confirming our previous evaluation of its prognostic and clinical phenotypic value. We also found that the expression of KLRB1 was positively correlated with lymphoid cells in almost all cancers. After analyzing the correlation between multiple immune signatures and KLRB1, we found that the expression level of KLRB1 in pan-cancer was the most consistent with TIL infiltration, in terms of immune CYT, HLA expression, and IFN response. Recently, several studies have identified a subset of CD161-expressing T cells in the TME [28, 30, 32, 40], and a single-cell RNA sequencing study pointed out that in SKCM, LUSC, LIHC, COAD, and GMB tumors, CD4+ T and CD8+ T cells all exhibit different levels of KLRB1 expression and CD161+ T cells can inhibit cytotoxicity and cytokine secretion [19]. The proportion of CD4+ T cells expressing CD161 in tumors has also been shown to be increased [41]. Furthermore, our GSEA findings showed that KLRB1 is highly enriched in the tumor T cell receptor signaling pathway, B cell receptor signaling pathway, antigen processing and presentation, and in other immune-related pathways. Altogether, our findings indicate that KLRB1 may influence tumor immunity mainly by mediating TILs.

At present, immunotherapy has become an emerging approach to cancer treatment, and significant progress has been achieved in some cancers [16]. However, many factors limit the efficacy of immunotherapy, and only some patients will obtain a good response [42, 43]. Therefore, it is extremely urgent to identify new immune targets and predictive biomarkers of prognosis. We found that compared with the existing predictive immune signal indicators TMB [33] and degree of glycolysis [26], KLRB1 expression was more strongly correlated with the immune score and cytotoxicity, suggesting that the expression of KLRB1 may be more representative of tumor immunity. In SKCM, the low expression of CD161 was found to reduce the cytotoxic activity of NK cells [44].

Similarly, in prostate cancer [18], non-small-cell lung cancer [15], and triple-negative breast cancer [45], the enhanced interaction between CD161 and its upstream molecule LLT1 inhibits the cytotoxic activity mediated by NK cells. Blocking the CLEC2D-CD161 pathway may be a potential target for immunotherapy in patients with diffuse glioma [19]. Moreover, CD8+CD161+ T cells have been proposed as cells with high therapeutic potential [40]. In this context, we further explored the relationship between KLRB1 and checkpoint genes and found a significantly positive correlation between KLRB1 expression and checkpoint gene expression in almost all cancers, which suggests that KLRB1 may have the ability to predict the response of patients to immunotherapy. Based on previous studies, the correlation between KLRB1 and TMB and MSI and the role of KLRB1 in TME, it is rational to speculate that KLRB1 has potential as a new immunotherapy target.

Chemotherapy is also an essential approach for clinical treatment of cancer, but its drug resistance and severe side effects have hindered the outcome of chemotherapy [27]. This study selected two broad-spectrum antitumor drugs, cisplatin and doxorubicin, and found that the expression of KLRB1 was closely related to drug sensitivity. For most tumors, those with low KLRB1 expression are more sensitive to chemotherapeutics. These results indicate that patients with low expression of KLRB1 may achieve better chemotherapeutic effects, which will help guide clinical drug selection and patient prognosis.

It is undeniable that there are some limitations in our research. Although we have used two different methods to evaluate TME, both were achieved through bioinformatics methods and as mentioned in the previous study [46] may have been influence by noise. We do not have specific drug efficacy data; thus, the evaluation of drug sensitivity was also based on the R package, so there were also absolute deviations. Finally, our research does not present data regarding immunotherapy. Therefore, the assessment of the ability of KLRB1 to predict tumor immunity and predict immunotherapy response is based on indirect evidence.

## 5. Conclusions

Our study provides strong evidence for the prognostic and immunological value of KLRB1 in various tumors through a comprehensive pan-cancer analysis. Our data indicate that KLRB1 is a protective gene in most cancers. We also found that KLRB1 may affect tumor immunity by affecting the levels of infiltrating immune-related cells, especially macrophages and lymphoid cells. Further studies are necessary to evaluate whether KLRB1 may be used as a new target for immunotherapy and has the potential value of predicting immunotherapy response and chemotherapeutic drug sensitivity. Our study will provide a better understanding of the molecular mechanisms involving KLRB1 in tumorigenesis and tumor development and provides a rationale for future immunotherapy and precision medicine.

## Data Availability

The RNA sequencing data and clinicopathological and survival data of 33 cancers were downloaded from UCSC Xena database (https://xena.ucsc.edu/). Tumor cell line's data were downloaded from the CCLE database (https://portals.broadinstitute.org/ccle/). KLRB1 expression in 31 various tissues was downloaded from GTEx (https://commonfund.nih.gov/GTEx). Immunohistochemistry images of TREM2 protein expression were downloaded from the Human Protein Atlas (HPA) (http://www.proteinatlas.org/). All the datasets were open access datasets.

## Supplementary Materials

Supplementary Materials Figure S1: the relationship between KLRB1 expression and patient prognosis. (A) Univariate Cox regression of KLRB1 expression for disease-free interval (DFI) in 33 cancers. (B) Univariate Cox regression of KLRB1 expression for progression-free interval (PFI) in 33 cancers. (C) The Kaplan–Meier curves of DFI in the low and high groups stratified by KLPB1 expression. (D) The Kaplan–Meier curves of PFI in the low and high groups stratified by KLPB1 expression. Figure S2: the relationship between TREM2 expression and tumor microenvironment (TME). (A) Six tumors with the highest correlation coefficients between KLRB1 expression and immune score. (B) Six tumors with the highest correlation coefficients between KLRB1 expression and stromal score. Supplementary Table S1: list of datasets. Supplementary Table S2: list of the gene sets. Supplementary Table S3: correlations of KLRB1 expression with tumor mutation burden (TMB) and tumor microsatellite instability (MSI) in 33 cancer types. Supplementary Table S4: correlations of KLRB1 expression with TME in 33 cancer types. Supplementary Table S5: correlations of KLRB1 expression with immune signatures in 33 cancer types. Supplementary Table S6: correlations of KLRB1 expression, tumor mutation burden (TMB), and tumor glycolytic activity with immune score and immune cytolytic activity in 33 cancer types.
