# Supplementary material for: Systematic Pan-Cancer Analysis of KLRB1 with Prognostic Value and Immunological Activity across Human Tumors
Source: J Immunol Res. 2022 Jan 3;2022:5254911. doi: 10.1155/2022/5254911 (PMC8749375; doi:10.1155/2022/5254911)
Supplement: Supplementary Materials — Figure S1: the relationship between KLRB1 expression and patient prognosis. (A) Univariate Cox regression of KLRB1 expression for disease-free interval (DFI) in 33 cancers. (B) Univariate Cox regression of KLRB1 expression for progression-free interval (PFI) in 33 cancers. (C) The Kaplan–Meier curves of DFI in the low and high groups stratified by KLPB1 expression. (D) The Kaplan–Meier curves of PFI in the low and high groups stratified by KLPB1 expression. Figure S2: the relationship between TREM2 expression and tumor microenvironment (TME). (A) Six tumors with the highest correlation coefficients between KLRB1 expression and immune score. (B) Six tumors with the highest correlation coefficients between KLRB1 expression and stromal score. Supplementary Table S1: list of datasets. Supplementary Table S2: list of the gene sets. Supplementary Table S3: correlations of KLRB1 expression with tumor mutation burden (TMB) and tumor microsatellite instability (MSI) in 33 cancer types. Supplementary Table S4: correlations of KLRB1 expression with TME in 33 cancer types. Supplementary Table S5: correlations of KLRB1 expression with immune signatures in 33 cancer types. Supplementary Table S6: correlations of KLRB1 expression, tumor mutation burden (TMB), and tumor glycolytic activity with immune score and immune cytolytic activity in 33 cancer types. [file 5254911.f1.zip › SupplementaryFigure.docx]

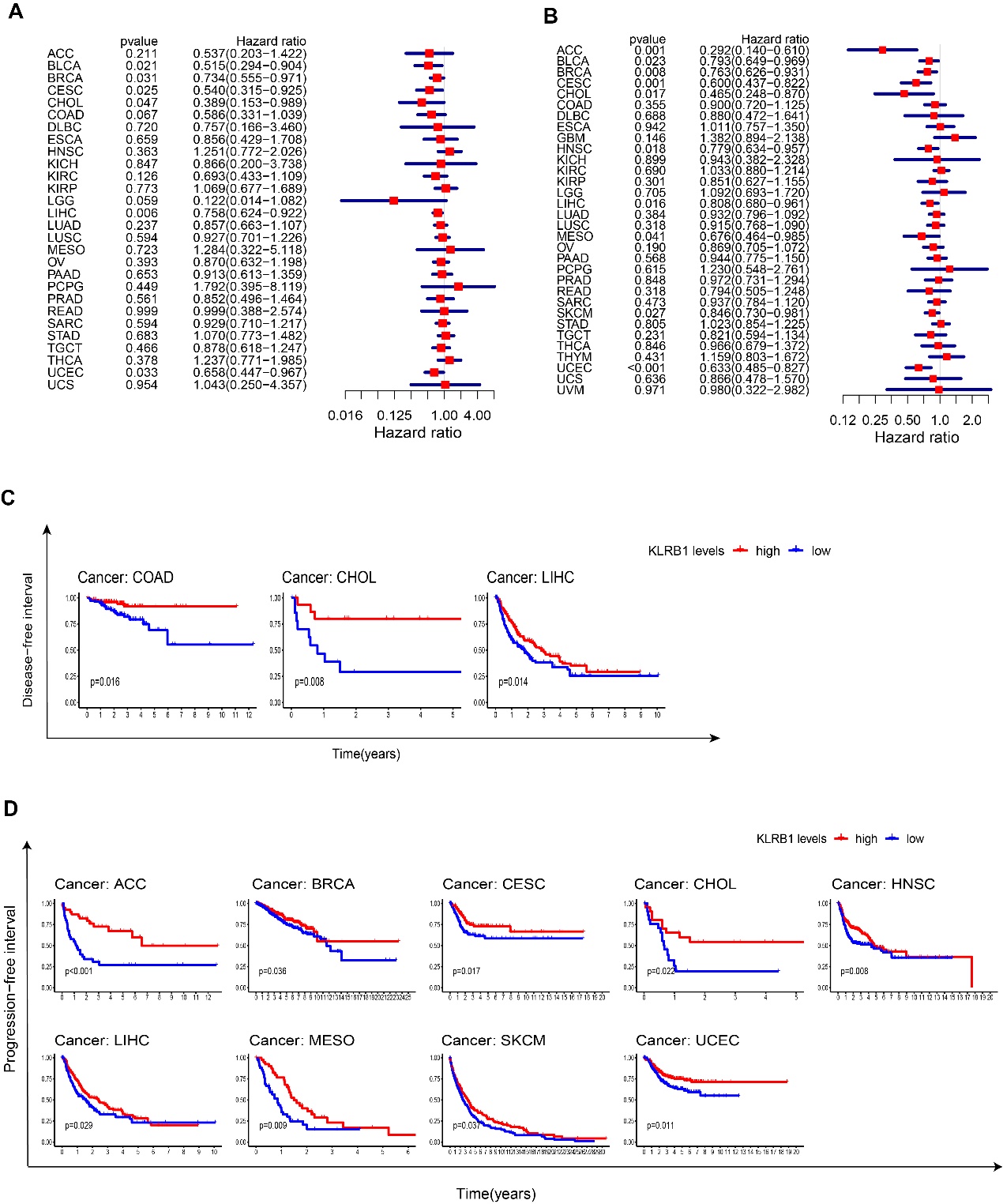


**Figure S1** The relationship between KLRB1 expression and patient prognosis. (A) Univariate cox regression of KLRB1 expression for disease-free interval (DFI) in 33 cancers. (B) Univariate cox regression of KLRB1 expression for progression-free interval (PFI) in 33 cancers. (C) The Kaplan–Meier curves of DFI in the low and high groups stratified by KLPB1 expression. (D) The Kaplan–Meier curves of PFI in the low and high groups stratified by KLPB1 expression.


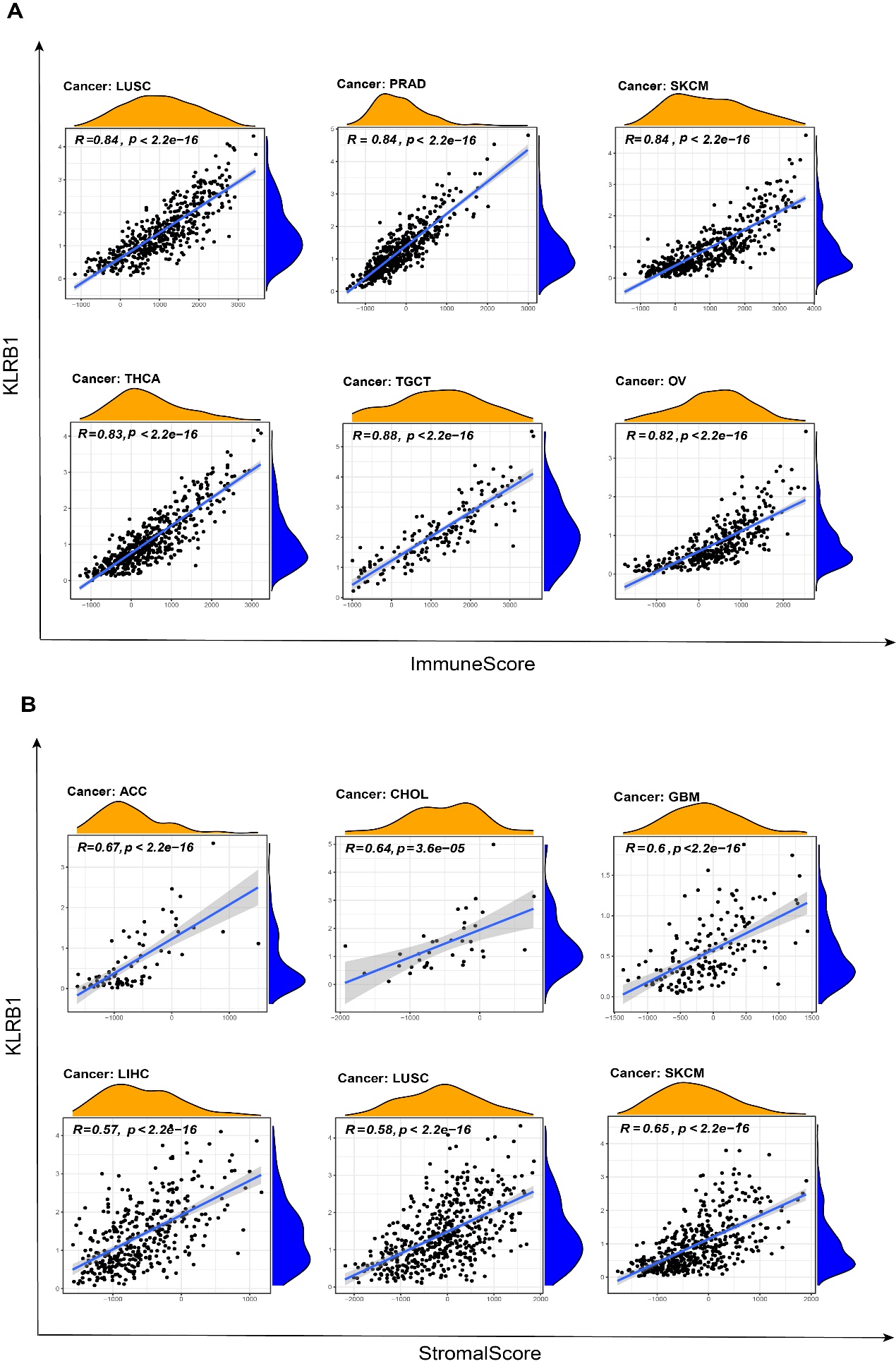


**Figure S2** The relationship between TREM2 expression and tumor microenvironment (TME). (A) Six tumors with the highest correlation coefficients between KLRB1 expression and immune score. (B) Six tumors with the highest correlation coefficients between KLRB1 expression and stromal score.
